# Additive Manufacturing of a Miniature Functional Trocar for Eye Surgery

**DOI:** 10.3389/fmedt.2022.842958

**Published:** 2022-02-17

**Authors:** Kirsten Lussenburg, Marta Scali, Aimée Sakes, Paul Breedveld

**Affiliations:** ^1^Bio-Inspired Technology Group (BITE), Department of BioMechanical Engineering, Faculty of Mechanical, Maritime, and Materials Engineering, Delft University of Technology, Delft, Netherlands; ^2^Dutch Ophthalmic Research Center International (DORC), Zuidland, Netherlands

**Keywords:** additive manufacturing, Stereolithography, miniaturization, ophthalmology, trocar, micro fabrication

## Abstract

Stereolithography is emerging as a promising additive manufacturing technology for a range of applications in the medical domain. However, for miniature, medical devices such as those used in ophthalmic surgery, a number of production challenges arise due to the small size of the components. In this work, we investigate the challenges of creating sub-millimeter features for a miniature, functional trocar using Stereolithography. The trocar cannula system is used in eye surgery to facilitate a passage for other instruments. A standard trocar consists of a hollow cannula and a flexible check valve. The research was performed in two stages: in the first stage we investigated the effect of different materials and print settings on the current design of the cannula and the valve separately, and in the second stage we used these findings to optimize the design and production process. After the first investigation, it became apparent that even though the dimensions of the trocar are within the feature size range of Stereolithography, all hollow features tended to fuse shut during printing. This effect appeared regardless of the materials or print settings, and can be attributed to refraction of the laser source. In order to circumvent this, we identified two potential strategies: (1) increasing the negative space surrounding features; and (2) decreasing the surface area per layer. By applying these strategies, we tested a new design for the cannula and valve and managed to 3D print a functional trocar, which was tested in an artificial eye. The design of the 3D printed trocar allows for further personalization depending on the specific requirements of both patient and surgeon. The proposed strategies can be applied to different applications to create miniature features using Stereolithography.

## Graphical Abstract

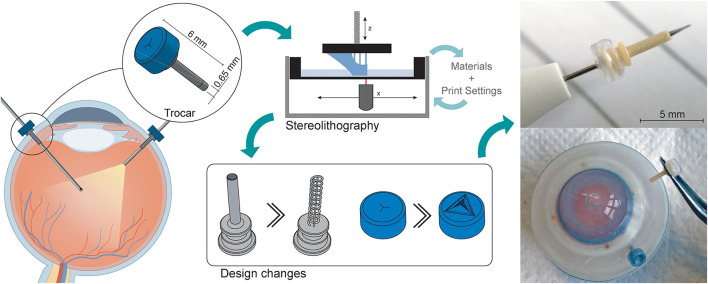

## Introduction

Additive manufacturing (AM) or 3D printing offers great flexibility in creating and producing complex parts and mechanisms. Especially in the medical domain, AM has been recognized for the benefits it offers in terms of personalization, additional functionalities, and production of increasingly complex structures (1, 2). In the field of ophthalmology, AM has been used for a variety of applications related to the eyes, such as the production of eye glasses, ocular prostheses, implants, and ophthalmic instruments, such as trocars (3–5). For ophthalmic surgical instruments, which are often in the sub-millimeter-scale range, it is important that AM technologies offer accuracy and repeatability, in addition to a small manufacturing size. Among the commercially available AM techniques, vat photopolymerization processes, such as Stereolithography (SLA) or Digital Light Processing (DLP), have become popular in the medical domain. Their popularity can be attributed to a high resolution, currently in the range of 25 μm (6–8), and the availability of biocompatible materials (6). Because of these factors, SLA and DLP are widely accepted in the medical domain, for applications ranging from patient specific implants to anatomical models (6, 9).

Despite the advantages of SLA and DLP techniques, when the size of parts or features approach the maximum resolution, limitations in accuracy become apparent. Manuals of 3D printers and technical datasheets of the materials only give theoretical information about the obtainable minimum accuracy, which does not always reflect the actual result. The final accuracy of a part is influenced by many factors, such as the material, process parameters and even the specific 3D printer (10). This means that it is likely that the 3D printed parts slightly deviate from the as-drawn dimensions. Therefore, for the design of miniature, functional devices, it is important to investigate the factors that influence the accuracy. In this research, we attempt to manufacture a miniature, functional trocar cannula system as used in ophthalmic surgery, while coping with minimal feature sizes of several micrometers and a high required accuracy (11).

### Trocar Design

**Figure 1:**
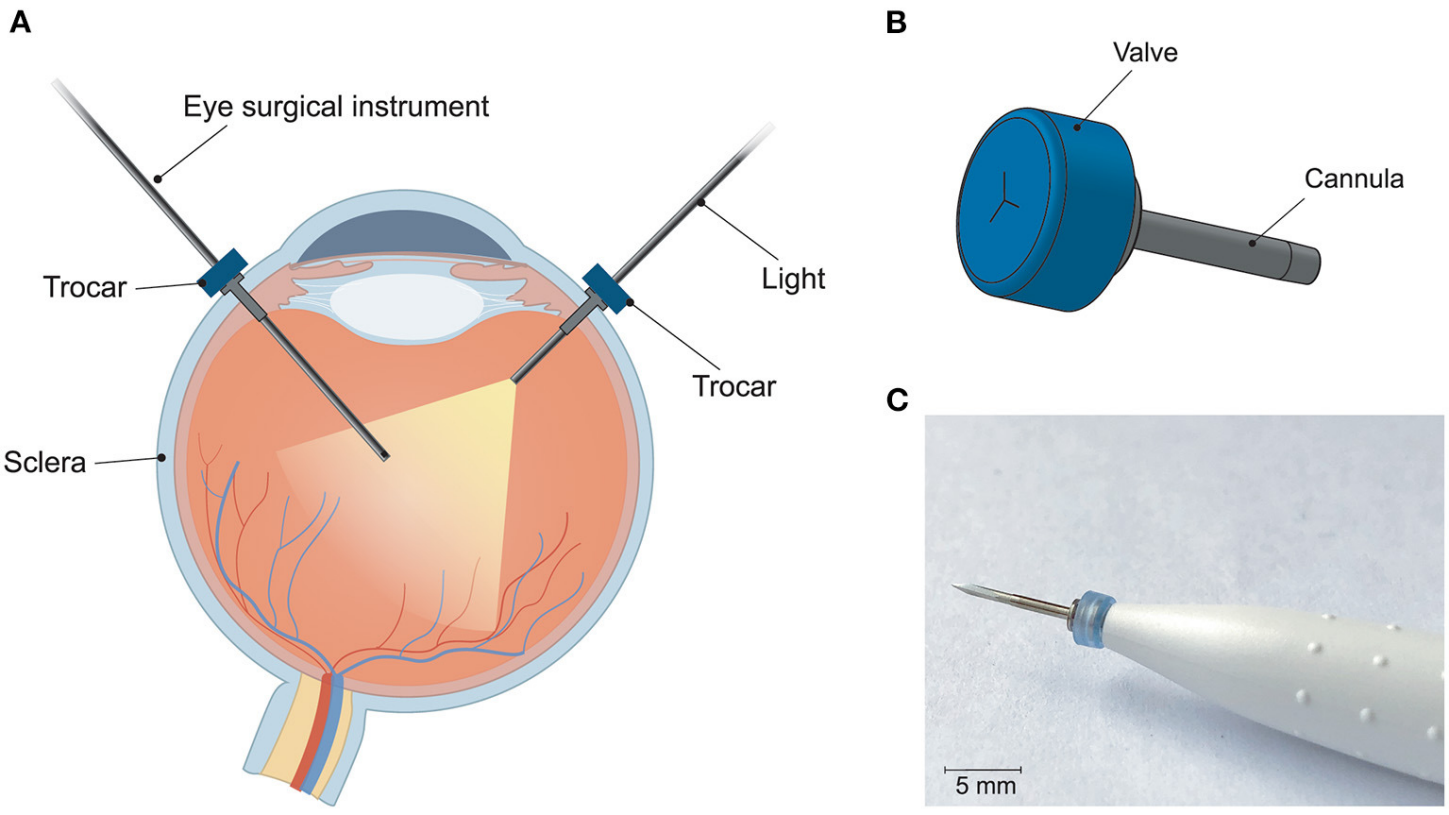
Trocar cannula system used in eye surgery, as developed by D.O.R.C. [Dutch Ophthalmic Research Center International (Zuidland, The Netherlands)]. **(A)** Schematic of a cross-section of the eye with a typical set-up for eye surgery. The trocars function as a channel into the eye for surgical instruments. Only the channel of the cannula is inserted into the eye, the top part of the cannula with the valve remains outside the eye; **(B)** schematic illustration of a trocar consisting of a valve (blue) and cannula (gray); **(C)** photograph of a trocar on the inserter knife, before insertion into the sclera.

The trocar cannula system is used in ophthalmic surgery to gain access to the interior section of the eye, by providing a working channel for other surgical instruments (Figure 1). The trocar is placed through the sclera, which is the outer protective layer of the eye, by means of a small incision made with an inserter knife. The trocar itself consists of a metal channel, the cannula, with a flexible valve on top (Figure 1B). After placement of the trocar, the channel is kept closed by the flexible closure valve. This is necessary in order to ensure that no fluids or gases are exiting the cannula, and to maintain a steady intraocular pressure during the surgery (11). The valve has three functions: (1) compliancy to let an instrument enter the eye, (2) enough springback to close after the instrument is removed without permanent deformation, and (3) no open gaps when the valve is closed to prevent leakage, which can be achieved by a distance of <10 μm between the valve flaps. The inner diameter of the channel must be compatible with the industry standard sizes of instruments used during ophthalmic surgery, which are commonly 23G (0.64 mm), 25G (0.51 mm) and 27G (0.40 mm) (12). Since other surgical instruments will be inserted through this channel, the internal accuracy is of greater importance than the outer accuracy, although a smaller outer diameter is preferred to minimize the size of the incision and the insertion forces. The length of the cannula channel is commonly around 4 mm, which is long enough to reach the internal cavity of the eye, but not too long to cause damage to internal structures (13).

### Stereolithography

The principle behind SLA is vat photopolymerization, in which a liquid photopolymer is exposed to a light source that cures the material into a final object. The light is generated by a single laser spot, the size of this spot determines the minimum feature size (14). The x,y-resolution is defined by the precision with which the laser spot can move, whereas the z-resolution is defined by the minimum layer thickness, i.e. the precision with which the z-axis can move. For this study, the SLA printer used was the Form 3B (FormLabs, USA), which has a reported x,y-resolution of 25 μm, and a minimum layer height of 25 μm (15). A single laser is used for curing, with a laser spot size of 85 μm, 405 nm wavelength, and 250 mW power (15). The FormLabs system is a closed system, which means that it is not possible to adjust settings such as laser power or scanning speed of the printer itself, rather the slicing software will decide on the appropriate settings based on the chosen material.

Although often used interchangeably, there is a difference between the resolution of a 3D printer and the dimensional accuracy it can produce (16). The resolution is a 3D printer-specific value, for which often the increments with which the laser spot can move are used, which can be used as an indication for the minimum feature size. The dimensional accuracy is dependent on many different factors, among which the material, laser intensity, calibration of the 3D printer, geometry of the part, color of the resin and even the settings of the file format used (16–18). Due to these many factors, it is difficult to give a general value for the dimensional accuracy of a specific AM technique (16). Previous studies have attempted to find the optimal process settings for increasing the accuracy of SLA processes, although it remains unlikely that these will be the same for all possible designs. The influence of process settings such as build orientations, printing direction, layer height, and material properties on the dimensional accuracy have been extensively studied (19–27).

The materials used in SLA are liquid photopolymer materials, engineered to cure under a light source. The exact composition of the materials is mostly proprietary information of the manufacturer, the materials are usually known by given names that describe one of their properties or applications. Little information is available on the mechanical properties of these resins, therefore selection of appropriate materials will often be on a trial-and-error basis. To remove residual liquid resin from the cured material after printing, the part is typically washed in isopropanol. Afterwards, a post-curing step takes place using heat and UV-light, to obtain the optimum mechanical properties of the material.

### Challenges

For the trocar, there are a number of challenges in its current design. The design of the cannula relies on the successful printing of a small, open channel, and for the valve three open incisions need to be printed accurately. These type of hollow features with small dimensions (>200 μm), i.e. negative features, proved difficult in the production of microneedles (28) and microfluidic devices (29, 30). Such enclosed channels are generally fused shut during the production process. In contrast, small, “positive” features can be produced accurately using SLA (31). To investigate these limitations, the design and printing of the trocars was carried out in two stages. In the first stage, the design was printed as-is with minimal changes, to determine the most suitable print settings and materials, as well as indicate problematic areas that require a redesign. In the second stage, the design and process are optimized for SLA printing, in order to obtain a functional trocar.

## Initial Design

### Initial Design and Method

**Figure 2:**
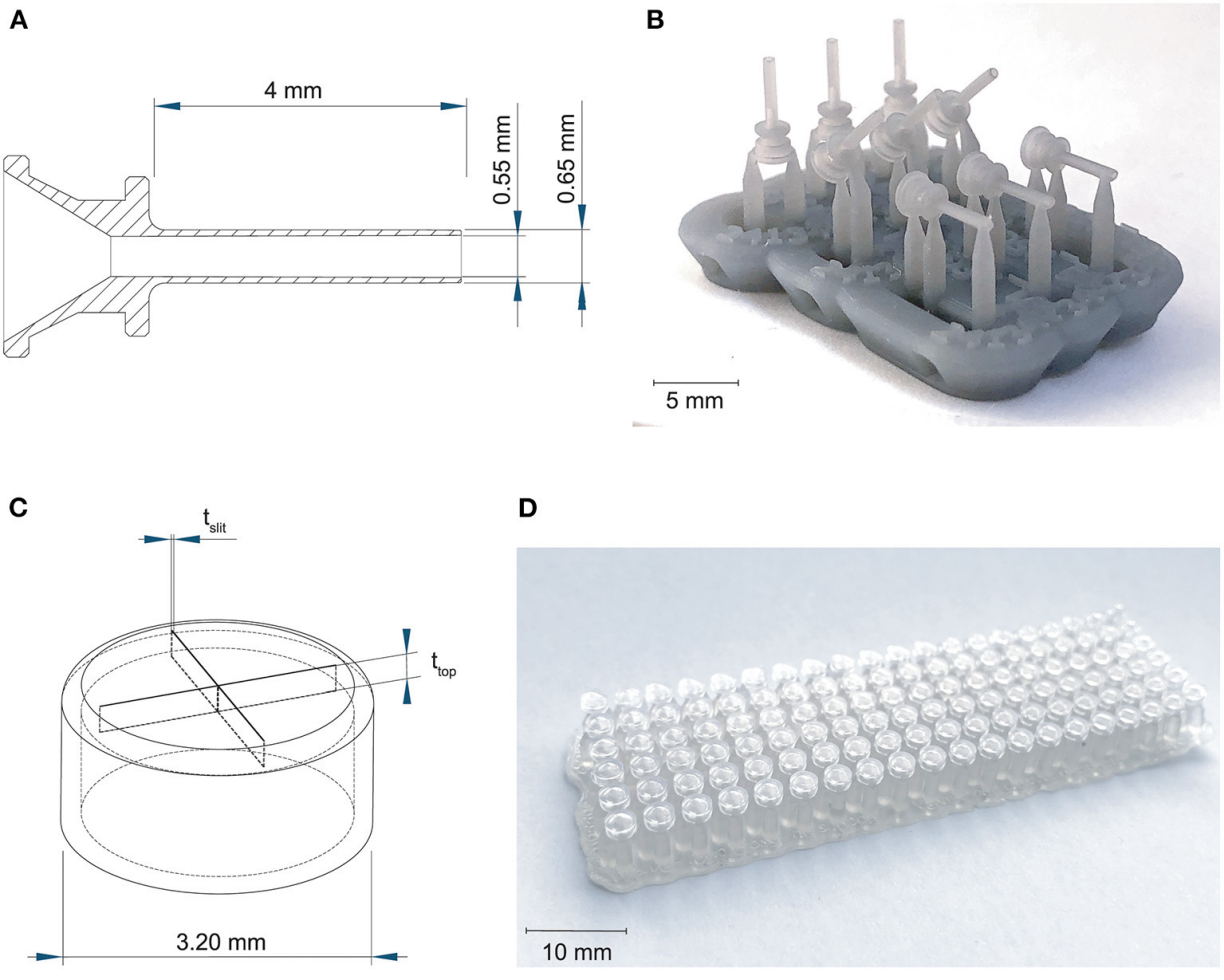
Overview of the 3D printed cannulas and valves. **(A)** Design and dimensions of the printed cannula; **(B)** example of the 3D printed cannulas in three different orientations, still on their support structure; **(C)** design and the dimensions that were tested for the valve, in which t_slit_ is the thickness of the slits, and t_top_ is the thickness of the top; **(D)** example of the 3D printed valves with different dimensions, still on their support structure.

First we investigated the effect of the used materials on the accuracy of positive and negative features of the trocar. The challenge in printing the cannula is obtaining an open channel of the desired dimensions with the thin wall thickness required (Figure 2A). Four materials were investigated to 3D print the cannulas: Dental SG, Model, Grey Pro, and Durable, of which Dental SG and Model are both biocompatible materials. In addition, two process settings were tested: (1) the build orientation and (2) the layer height. The cannulas were printed in three different print orientations: 0°, in which the channel is positioned vertically, 45°, and 90°, in which the channel is positioned horizontally (Figure 2B). For the layer height, the smallest and largest layer heights were chosen. Depending on the material, the smallest layer height was 25 or 50 μm, while the largest layer height was 100 μm for all materials. For each print setting three cannulas were printed, resulting in a total of 18 printed cannulas per material. After printing the parts were washed and post-cured according to manufacturer instructions, and measurements were taken using a digital microscope.

The challenge in 3D printing the valve is to obtain separate valve flaps that can move independently, but are close enough together to prevent leakage. To facilitate this, we adjusted the design to be more suitable for 3D printing by maximizing the length of the slits to 2.6 mm, which is the maximum length without changing the size of the cap itself, and by applying a four slit cross shape instead of the three slits in the original design in Figure 1B, to allow for more compliancy. The tested parameters were the top layer thickness and the slit thickness, illustrated in Figure 2C, since both influence the functions of the valve. The thickness of the top layer (t_top_) was tested in thicknesses starting at 75 up to 200 μm, with increments of 25 μm. The thickness of the cuts of the slits (t_slit_) ranged from 10 to 200 μm, with increments of 10 μm. The assumption here is that for the sizes larger than the desired slit size, the slits will be partially fused and end up approaching the desired slit size. Based on these dimensions, a total of 120 valves were printed per material (Figure 2D).

Four different materials were investigated to 3D print the valves: Dental SG, Durable, Elastic 50A, and Flexible 80A. A digital microscope was used to measure the *t*_*slit*_ of the valves for which the slits were as close together as possible, but not fused shut. Subsequently, a 27G hollow needle was used to test the valves' compliancy and level of springback, by checking if the needle could be inserted without breaking the valve (level of compliancy) and if after the removal the valve could return to its initial position (level of springback).

### Initial Design Results

**Figure 3:**
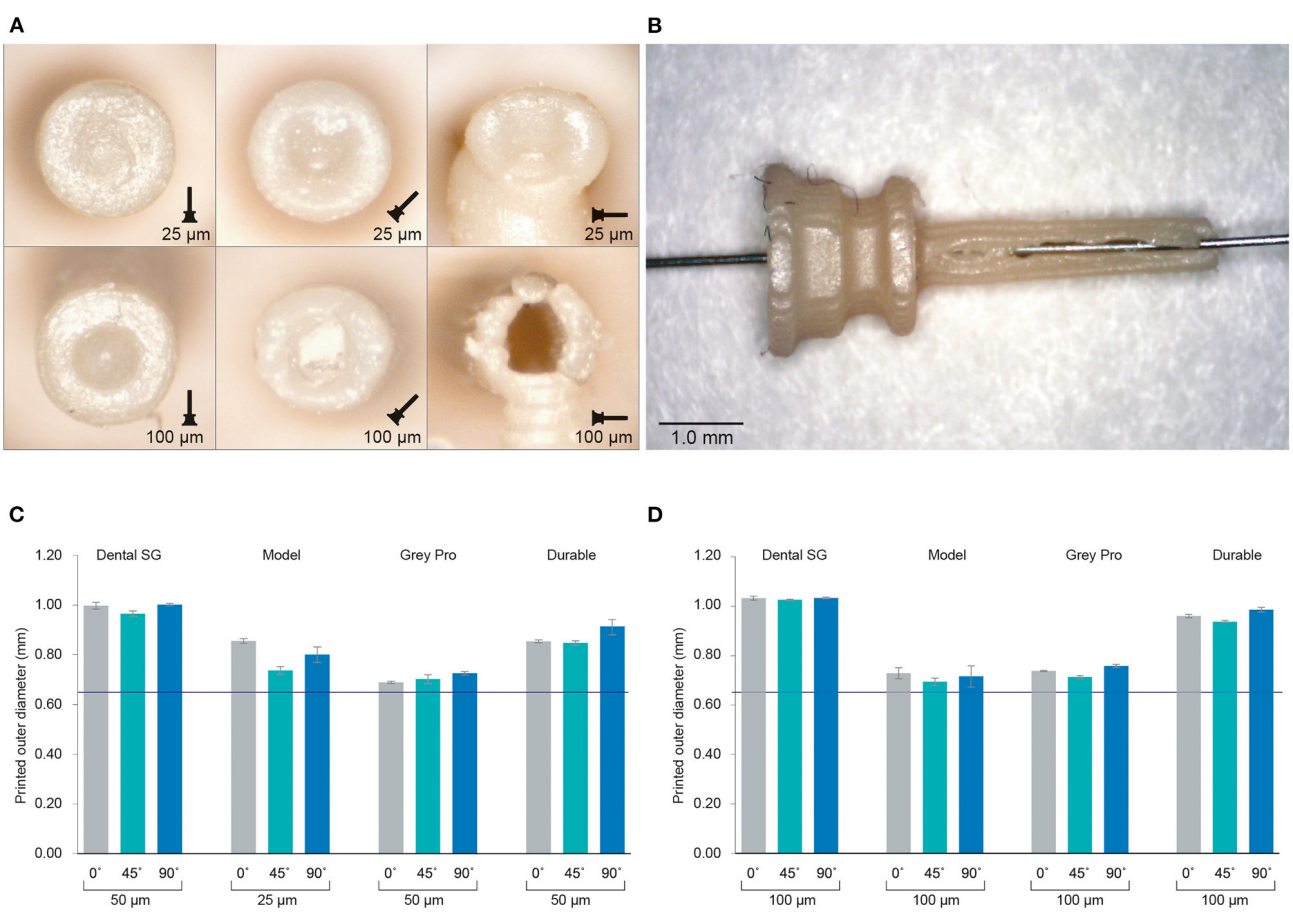
Results of the investigation into different materials and print settings for the cannulas. **(A)** Example of the distal ends of the valves printed in Model, which was among the most successful materials, in different orientations and with different layer heights. For the cannulas in the 90° orientation, the support pillar is visible at the bottom. **(B)** Cannula printed in Model with 100 μm layer height and open internal channel, although the wall is visibly torn. **(C)** The average outer dimensions of the channel given per material and per build angle for the smallest layer height setting per material. The purple line indicates the as-drawn dimensions of the outer channel. **(D)** The average outer dimensions of the channel given per material and per build angle for the largest layer height setting per material. The purple line indicates the as-drawn dimensions of the outer channel.

The inner channels of the 3D printed cannulas were in almost all cases fused shut, regardless of material, layer height or build angle, with only an indentation visible at the distal end of the cannula (Figure 3A; Supplementary Figure 1). Only the cannulas printed with Model with a layer height of 100 μm in the 90° orientation had a visibly open channel. However, the walls were very fragile and tore immediately when inserting a hollow needle (Figure 3B). In the 90° orientation, for all other settings and materials, the cannulas had no open channel and were severely deformed. Since most of the cannulas were fused shut, only the dimensions of the outer channel were measured, for which the results are given in Figures 3C,D; Supplementary Table 1. When compared to the as-drawn outer dimensions of the cannula (purple line in Figures 3C,D), Dental SG has the lowest accuracy for all print settings, while Model and Grey Pro have the highest accuracy. There appear to be no significant differences between the different orientation angles, although the 90° orientation shows a slightly lower accuracy for all materials.

**Figure 4:**
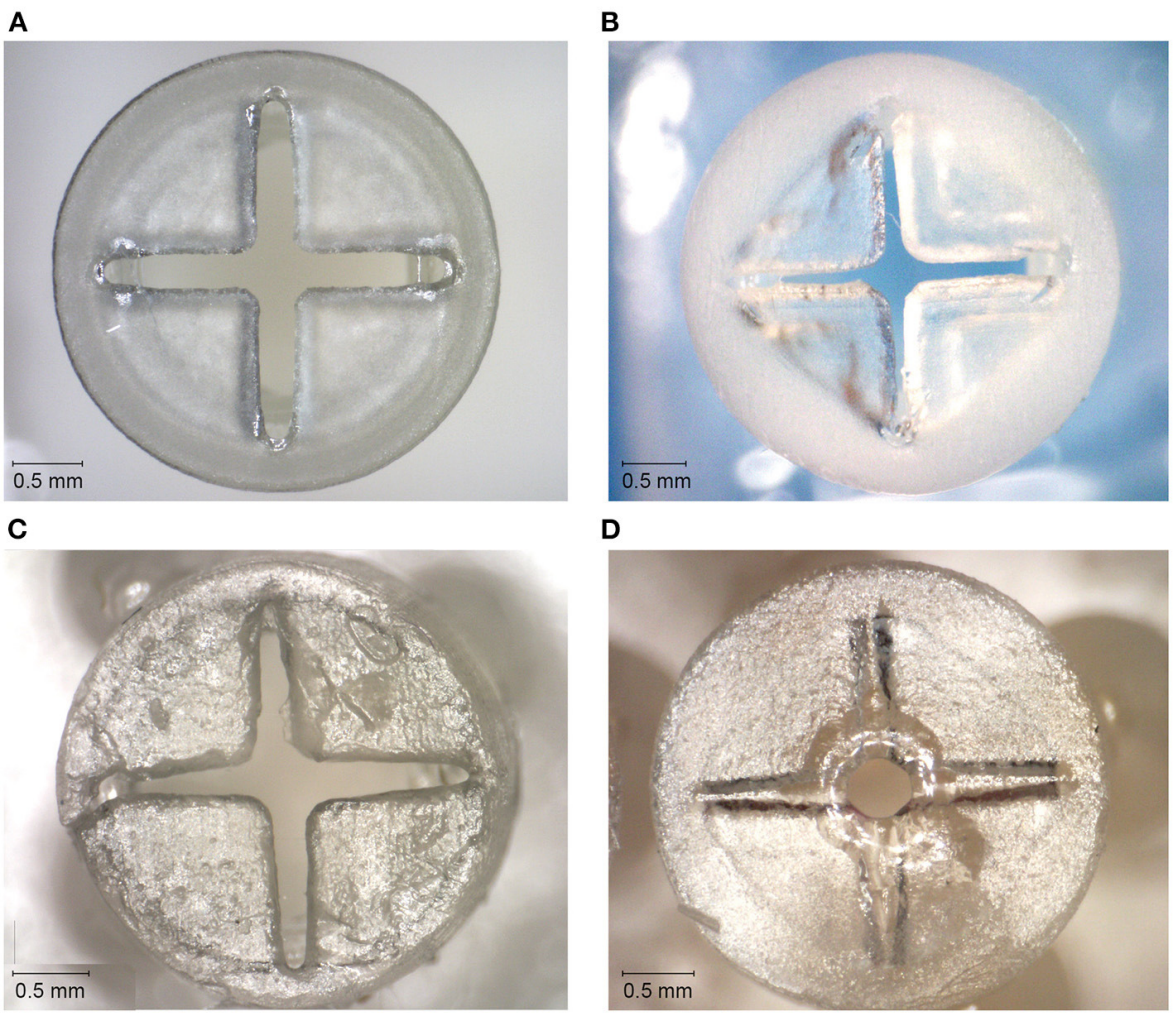
Top surfaces of the 3D printed valves with the smallest top-and slit dimensions for different materials. **(A)** Valve printed in Dental SG. **(B)** Valve printed in Durable. **(C)** Valve printed in Elastic 50A. **(D)** Valve printed in Flexible 80A, with only an opening in the center of the valve.

The valves with the smallest top- and slit dimensions, for which the valve flaps were not fused together, were measured for Dental SG, Durable, and Elastic 50A (Table 1; Figure 4), and their negative surface area was calculated (Supplementary Figure 2). The valves printed in Flexible 80A were all completely fused, except for a hole in the center (Figure 4A). For the other materials, it was noticeable that although the printed openings of the slits were larger than drawn, a 10 μm increment smaller in drawn slit size would mean that the slits were partly or fully fused together. A possible explanation for this is material shrinkage, which can more easily occur when the valve flaps are not attached to each other. Insertion of the hollow needle into the valves showed that the materials Dental SG and Durable were too brittle, resulting in breakage of the valve flaps rather than deformation (Supplementary Figure 3A). Both Elastic 50A and Flexible 80A showed compliant behavior and flexibility (Supplementary Figure 3B), although Elastic 50A was so soft that it was easy to puncture.

**Table 1:** Dimensions of the valves with the smallest top-and slit dimensions.

	**Dental SG**	**Durable**	**Elastic 50A**	**Flexible 80A**
Slit thickness as-drawn	140 μm	90 μm	150 μm	200 μm
Top thickness as-drawn	75 μm	75 μm	200 μm	75 μm
Printed slit thickness (max.)	248 μm	131 μm	261 μm	-

*The printed slit thicknesses were measured using a digital microscope*.

### Initial Design Discussion

Based on the 3D printing experiments, the design of the trocar as-is is incompatible with SLA, regardless of the material or print settings. We compared different materials, build orientations and layer heights for the cannula, but none resulted in a usable, opened internal channel. The measurements of the outer diameter showed that in all cases the cannulas were printed larger than drawn. Model and Grey Pro approached the drawn dimensions the closest, in which Model has the advantage of being biocompatible. Some studies have indicated that a higher layer thickness leads to a more accurate part (24, 25), however in our case the size of the layers relative to the part was too large to be functional. The build angles of 0° and 45° showed most promising results. The ones printed in 90° were more oval than round, and needed extra support pillars, which would cause the cannulas to break when removing them. Although multiple studies have researched factors that can improve the accuracy, our study shows that these can be conflicting for the purpose of a functional product. For instance, for the cannulas printed in Model, it was possible to print an open channel with a higher layer thickness. However, a higher layer thickness also resulted in cannula walls that were too fragile, which resulted in a non-usable cannula.

None of the valves were printed with the required 10 μm slits thickness. In the cases the slits were not fused together, they were larger than drawn, and therefore not usable as a valve. A possible solution could be to use the valves that were mostly fused shut, since these have a lower percentage of open surface area. However, as was seen in the compliancy test, this will lead to breakage of the valve when inserting an instrument. For Elastic 50A and Flexible 80A, which have a high flexibility, the hollow needle was able to puncture the top surface, however this resulted in a hole in the valve that cannot be closed off. Instruments compatible with this size of trocar have a diameter of 460 μm, which is only slightly smaller than the diameter of the internal channel of the cannula (550 μm), which would not be able to prevent leakage.

As previous studies have pointed out, refraction and diffraction of the laser spot can lead to an effect sometimes referred to as “false printing” (29, 32), in which material outside of the laser spot is slightly cured. When printing a hollow tube, such as the cannula, the laser spot draws a circular shape around a negative space for each layer. Due to the small size of this negative space, the cumulative effect of false printing builds up and fully cures the internal channel as well. One option to obtain a functional trocar would therefore be to adjust the feature the size of the cannula, for instance to 20G (0.81 mm). However, this size of trocar has been abandoned in eye surgery in favor of smaller sizes. In an attempt to circumvent the false printing effect and stretch the limits of the printing technology, we propose two solutions: (1) increase the size of the negative space surrounding the features; (2) decrease the curable surface area of each layer, and make sure this surface area is not in the same spot for each layer. Using these solutions, in the next section we present a new design for the cannula and the valve specified to the SLA process.

## Design Optimization

### Optimized Design and Method

**Figure 5:**
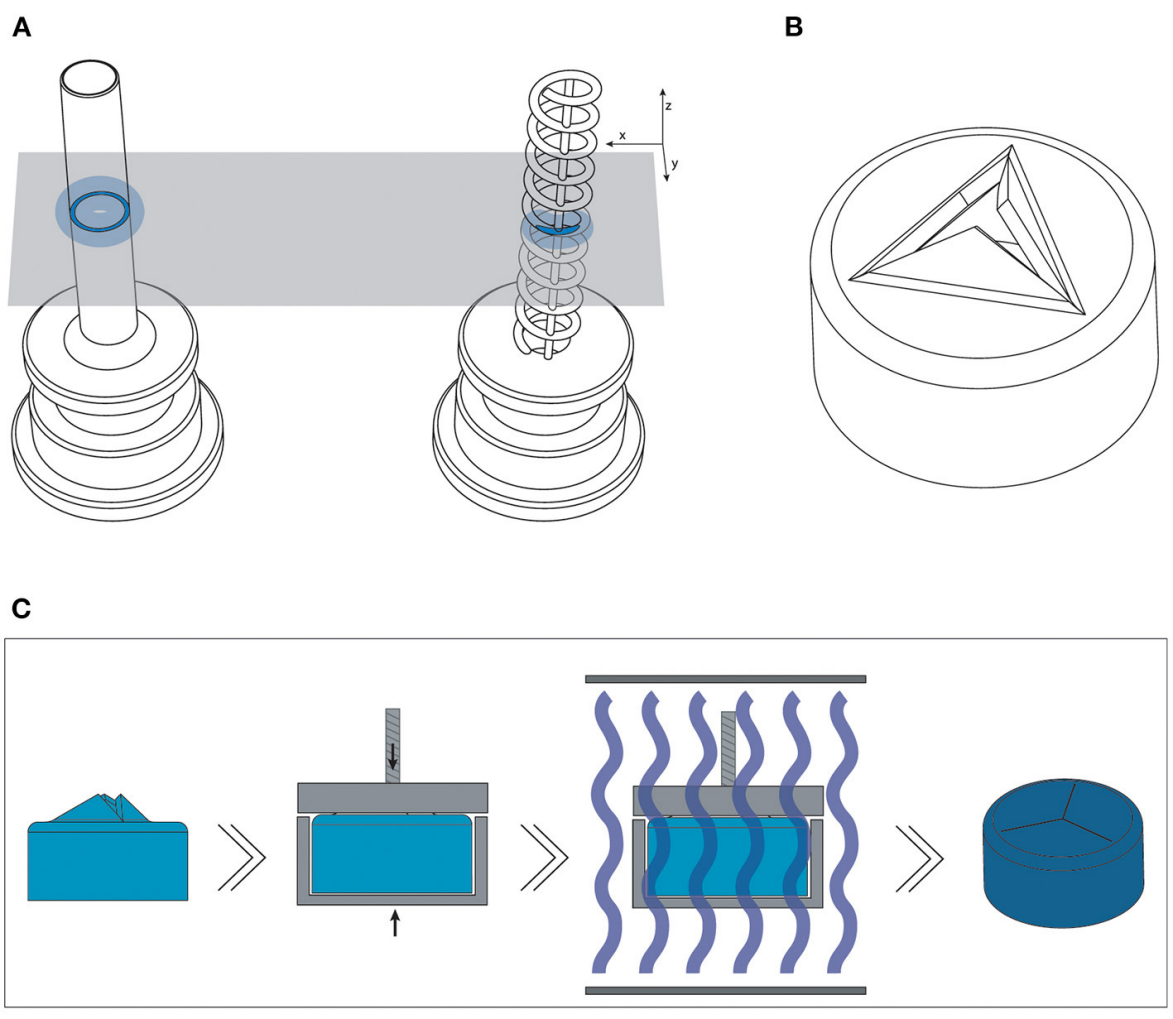
Overview of the optimized design and method for printing the trocars. **(A)** The new helix design (right) of the cannula, which has a smaller surface area (dark blue) per layer than the previous cannula (left), and should therefore be less hindered by the effects of false printing (light blue). **(B)** 3D model of the valve, in which the valve flaps are modeled to point upwards in order to create more negative space around them. **(C)** New production process steps for the valves: after printing and cleaning, the valves are placed in a clamp which presses the flaps down; the entire clamp and valve are subsequently cured.

To optimize the cannula for 3D printing, we explored the possibility of printing a trocar with a minimum curable surface area per layer and maximization of the negative space surrounding it. In our design, the cannula is composed of a helical structure (Figure 5A), so that each layer has a smaller surface area that needs to be cured, as compared to that of a hollow tube. In addition, considering the structure is printed in the vertical orientation, the to-be-cured surface area is not in exactly the same spot for each layer, which results in less cumulative build-up of false printing. The thickness of the helix, which is the wall thickness, is 100 μm, equal to the wall thickness. Although the original cannula design has a wall thickness of 50 μm, this is smaller than the size of the laser spot (85 μm), and can therefore not be produced in this size. Two vertical columns with the same thickness were added to function as custom support structure, to ensure printability, and to prevent the structure from acting as a spring. To prevent the sides of the channel from being completely open, the pitch of the helix should be as small as possible, so that the windings of the helix will fuse together. To find the optimal value, we tested the helix with a pitch of 0.40 mm (10 revolutions), 0.33 mm (12 revolutions), 0.29 mm (14 revolutions), 0.25 mm (16 revolutions), 0.22 mm (18 revolutions), and 0.20 mm (20 revolutions). The new design was printed in Model, since this material showed accurate results in the previous tests and is biocompatible, in the vertical 0° orientation with a layer height of 50 μm. After printing the parts were washed and post-cured. Measurements were taken with a digital microscope and with a Scanning Electron Microscope (SEM) of the internal lumen and the wall thickness.

For the valve, we attempted to create more negative space surrounding the miniature features. We accomplished this by opening up the flexible flaps in the CAD file, so that they are positioned upwards (Figure 5B). This creates more negative space surrounding the flaps, while maintaining the right dimensions. Ideally, the flaps would be printed in a downward open position, so that the pressure in the eye causes them to close, however the limited space in the cap of the valve makes it impossible to print them separately from each other in that position.

The flaps were opened under an angle of 60°, with a flap- and top thickness of 0.15 mm. In order to negate the effects of shrinkage, the flaps were designed to be slightly longer than required when printing them flat, 0.69 mm instead of 0.54 mm, which was experimentally determined after a number of try-outs. The valves were printed in Flexible 80A, which showed the best compliant behavior in our previous test, with a layer thickness of 50 μm. In the post-curing step after printing and cleaning, the flaps were pressed into their correct closed position, by placing them into an enclosure with a transparent lid. The entire enclosure was then cured, and after cooling the flaps remained in the desired, closed position. This process is schematically shown in Figure 5C. Measurements were taken with a digital microscope and with a Scanning Electron Microscope (SEM). The tests with insertion of the needle were repeated to determine their compliancy and springback.

### Optimized Design Results

**Figure 6:**
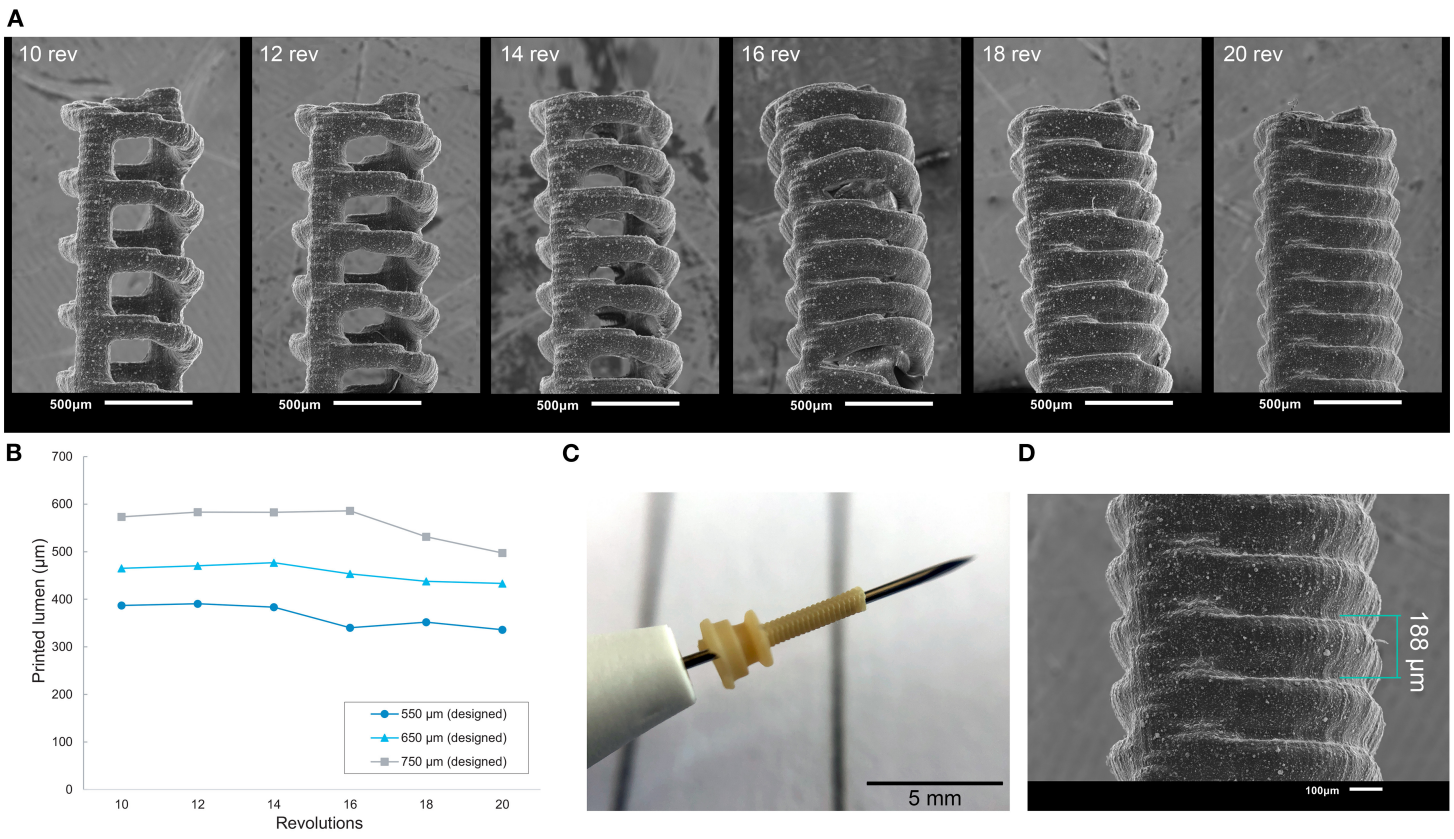
Results of the new cannula design. **(A)** SEM images of the tips of the cannulas showing the effects of different numbers of revolutions of the helix. **(B)** Influence of the number of revolutions on the size of the internal channel of the cannulas. **(C)** close up of a 3D printed helix cannula placed on the tip of a 27G inserter knife. **(D)** SEM image and measurements of the helix cannula with 20 revolutions.

The new design of the cannula prevents false printing from fusing the internal lumen shut. With this design, all cannulas were printed with an opened internal lumen. The internal lumen was measured with a digital microscope, and the thickness of the printed helix with a SEM. In order to be used in eye surgery, the cannula should accommodate instruments of standard size, which can be 400 μm at minimum. Therefore, the inner lumen of the trocar should be slightly larger than this size. As depicted in Figure 6B, the number of revolutions of the helix, and therefore the size of the pitch, influences the internal lumen of the cannula: the more revolutions, the smaller the remaining lumen. The difference between the printed diameter and the drawn diameter increased from 175 μm on average with 10 revolutions, to 228 μm on average with 20 revolutions. As can be seen from this graph, in order to obtain a lumen of more than 400 μm, the helix should be designed with an inner diameter of 650 μm. This result is consistent with our approach of decreasing the size of the positive features, since more revolutions decrease the distance between the windings, and result in a larger surface area per layer. However, too little revolutions and there is spacing between the revolutions (Figure 6A), which is also undesirable for our purpose. The helix with 20 revolutions shows no gaps in the side of the channel (Figures 6C,D), and is therefore most suited for the cannula. The outer diameter of these cannulas was measured to be 828 ± 13 μm. It should be noted that with these settings the size of the inner diameter corresponds to a 27G trocar size, while the size of the outer diameter corresponds to the 23G trocar size.

**Figure 7:**
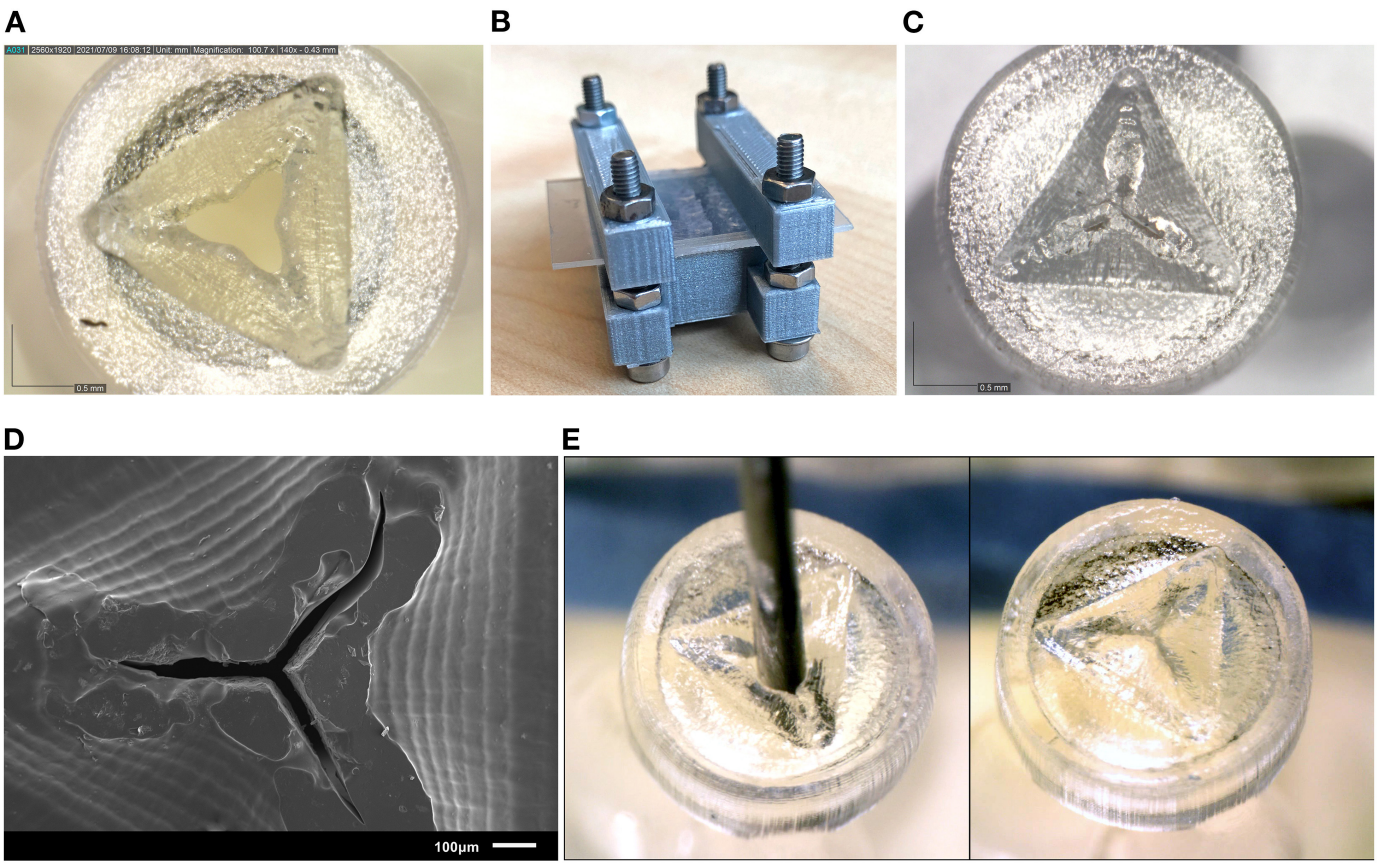
Results of the new valve 3D print process. **(A)** Close up of a valve before curing, showing the opened valve flaps. **(B)** Clamp set up for curing the valves. **(C)** Close up of the valve after curing, the valve flaps have been pressed to their horizontal position, and no gap is visible between the flaps. **(D)** SEM image of the valve after curing. **(E)** Needle insertion test of the new valves, the needle can be easily inserted through the valve (left) and afterwards the flaps return to their horizontal position (right).

The valves printed with the new process steps are shown in Figure 7. Measuring the slits in the valves using SEM (Figure 7D) shows that they range from 17 to 35 μm thickness. Although this is larger than the desired value of 10 μm, the open slits are not as long as in the original design, resulting in a smaller open surface area. The tests with insertion of the needle were also repeated, which showed that the material has enough compliancy to allow an instrument to enter, and the flaps to return to their closed position when the needle is removed (Figure 7E).

## Eye Phantom Insertion

### Insertion Test Method

**Figure 8:**
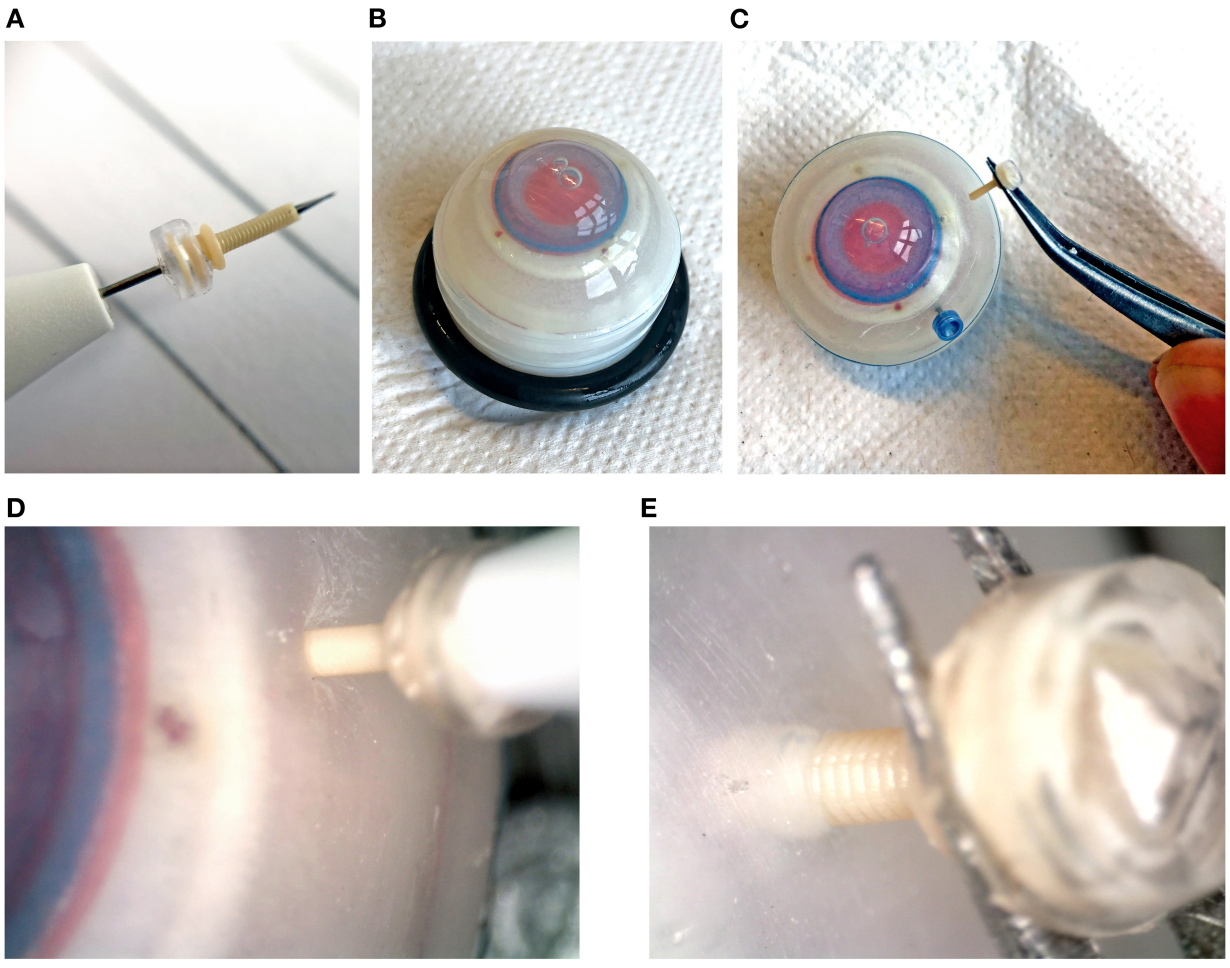
Insertion tests into an artificial eye phantom. **(A)** Assembled trocar on a 27G commercial inserter knife. **(B)** Artificial eye for testing. **(C)** Removal of the 3D printed trocar from the eye, showing that it is still intact. **(D)** Magnification of the insertion of the 3D printed trocar, showing the deformation of the sclera. **(E)** Magnification of the removal of the 3D printed trocar.

The use of a polymer instead of metal to produce the cannula results in an inherently weaker design. This could mean that the cannula will break during insertion into the eye. The performance of the 3D printed trocar was tested by inserting it into an eye phantom (Eyecre.at GmbH, Ötztal, Austria), while measuring the insertion force, and comparing it with a commercially available trocar. The phantom eye was fixed onto a digital precision scale. The 3D printed trocar was assembled by placing the valve on top of the cannula, after which it was placed on the knife of a commercially available 27G trocar inserter (D.O.R.C., Zuidland, The Netherlands). The trocar was inserted into the scleral phantom by means of a single step, straight insertion. After insertion, a digital microscope was used to check whether there was no leakage of fluids from the valve. Subsequently, the trocars were removed using tweezers and examined under a digital microscope for signs of breakage. The test was performed with five 3D printed trocars. The maximum force as measured by the digital scale was recorded for each insertion test. Images of the test are shown in Figure 8. For comparison, commercially available 23G and 27G trocars (D.O.R.C., Zuidland, The Netherlands) were inserted in the same manner (*n* = 5).

### Insertion Test Results

The maximum force measured during insertion for each of the trocars is shown in Table 2. The insertion force for the 3D printed trocar was almost 70% higher than for the commercially available 27G trocar, and almost 50% higher than for the 23G trocar. The higher force can be attributed to the larger wall thickness, which results in a larger step-difference between the knife inserter and the trocar, as well as the “ribbed” outer wall that results from the helix design. It was visible as a larger deformation of the scleral phantom (Figure 8D), while the commercial trocars showed almost no deformation of the phantom. None of the 3D printed trocars broke during insertion or extraction. The valves did not show leakage while in the eye, although no additional pressure was applied. In eye surgery, the eye is artificially kept on a certain pressure, which can range from 0 to 120 mmHg, depending on the specific operation (33), however for higher pressures it is often required that the valve allows fluid to escape (13).

**Table 2:** Maximum forces measured during insertion of commercial and 3D printed trocars into an artificial eye phantom.

	**Commercial 23G**	**Commercial 27G**	**3D printed 27G**
Maximum insertion force (N) (n = 5)	3.20 ± 0.37	2.82 ± 0.22	4.78 ± 0.26

## Discussion

### Process and Method

Functional complexity is often considered one of the main advantages of 3D printing. The ability to produce a complete, functional product in a single production step can have many advantages for the manufacturing process. However, functional complexity does not always come easily. In this study, we aimed to investigate whether it would be possible to produce a trocar used in ophthalmology using 3D printing. SLA was chosen as best suited 3D printing technique for this study because is known for its ability to produce small features and the wide range of materials available. However, the theoretical information available about 3D printing techniques and materials does not present a full spectrum of what is possible with a certain 3D printing technique. Therefore, first we investigated the suitability of different materials and process settings for both the cannula and the valve. Materials with different properties were used, in order to gain more knowledge on the minimum feature size achievable. Even within one 3D printing technology we have seen large differences between the accuracy and functionality that can be obtained for different materials. The main conclusion we can draw from this is that it is important for any successful 3D print design to be aware of the influence of the material choice in advance, therefore starting the design process with an investigation into these settings is recommended.

We succeeded in creating a functional trocar by circumventing the inherent limitations of the SLA process. The process we followed in order to accomplish this was (1) creating a thorough understanding of the challenges of the process; (2) exploring different materials and print settings to find the most suitable ones; (3) evaluating and adjusting which parts of the design still posed challenges for the chosen AM technology. This systematic approach for finding the relevant print settings, materials, and dimensions can be applied to any print method. We proposed two strategies, increasing the surrounding negative space and decreasing the surface area per layer, which are specific for SLA and circumvent the effects of false printing. Although these solutions were specific to our design and desired application, they have the potential for application in different fields for which miniature negative features are required. Increasing the surrounding negative space can be applied as a general strategy by folding the part open as we have shown here, dividing the part in different substructures, or even considering different build angles. Decreasing the surface area per layer can be applied by adjusting the structure as we have shown here, as well as adjusting the build angle. It should be noted that these changes may negatively affect the mechanical properties of the part, so a careful consideration is required.

We have investigated the effects of materials, build angles, and layer heights on the accuracy and functionality of our design. There are more factors that have been shown to influence the accuracy of SLA parts, such as exposure time and laser intensity (14, 34), that we have not investigated. Since the used Form 3B printer is a closed system, it is not possible to change these printing parameters manually. The slicing software will determine the optimal printing parameters based on the material and layer height that are chosen. A disadvantage is that it is not possible to adjust these parameters to improve the accuracy of the results. However, the advantage of the closed system is that the printer manufacturer can optimized the materials and settings for specific applications, as has been happening for dental applications in recent years, which improves the ease-of-use for users. This has caused the FormLabs system to be widely accepted in commercial dental practices, and therefore we consider this a realistic scenario for further integration of 3D printing technology and healthcare.

### 3D Printed Trocar

The 3D printed trocar does have some inherent drawbacks caused by the changes to the design. The wall thickness is larger than in the original design, which is caused by the minimum feature size of the 3D printing process. In addition, the ribbed wall from the helical structure might cause damage in the eye. To prevent this, an extra processing step could be added to smooth out the outer wall, or alternatively, a rectangular cross-section for the helix could be explored. Regardless, the tests in the phantom eye show that the plastic cannula has enough stiffness to be inserted and extracted into the sclera without breaking. This shows the feasibility of using a 3D printed trocar in ophthalmic surgeries, although more tests in for instance porcine eyes are required to determine the behavior of the trocar in actual tissue.

An additional challenge when producing miniature hollow features using SLA, is leftover resin becoming trapped (35), which we encountered when attempting to 3D print the initial design of the valve. In the new design of the valve, this was no longer a problem, since we opened up the valve flaps, creating sufficient space to clean out all excess resin. Although we added an additional process step in order to produce the valve by having to close the valve flaps, the advantage of better cleaning makes the process easier and leads to an improved end result. The same is true for the helical design of the cannulas. The design we used for testing with 20 revolutions showed that there was no spacing between the revolutions, however even with less revolutions it was possible to successfully print the design. The openness of the structure with less revolutions makes it more accessible to clean, which could also have contributed to the fact that less revolutions lead to a larger internal diameter.

The design of the 3D printed trocar allows for further personalization depending on the specific requirements of both patient and surgeon. For instance, the length of the cannula can be easily tailored to the thickness of the individual sclera. The thickness of the sclera can range from 0.39 to 0.67 mm (36), and it is important that the cannula is not too long to avoid damaging internal structures in the eye, yet long enough to reach the vitreous cavity. The design of the valve can be optimized to allow escape of fluids for certain pressures, by varying the length and thickness of the valve flaps. In addition, the trocars placed in the eye often have specific functions during the surgery, and AM enables that they can be adjusted for their specific function.

So far in this research, we have 3D printed a cannula and valve separately. In future work, it will be interesting to look into the additional benefit of 3D printing an entire assembly in one single production step. For the trocar, this would mean printing the cannula and valve as one part. Current commercially available SLA printers are limited to the production of only one material at a time, although multi-material SLA printers are in development (37). For now, this means that both parts of the trocar need to be made from the same material. Since the cannula requires a certain stiffness, the best suggestion would be a redesign of the valve to work with a stiffer material, for instance a duckbill valve. Alternatively, other techniques, such as PolyJet printing, can be explored, in which it is possible to combine rigid and flexible materials in a single production step.

## Conclusion

In this study, we attempted to 3D print a functional trocar intended for use in eye surgery, consisting of a hollow, stiff cannula, and a flexible valve. The trocar contains sub-millimeter scale features, which is why SLA was chosen as the preferred 3D printing technique due to the high reported resolution and minimum feature size. We divided the research into two stages; in the first stage we investigated the effects of different materials and print settings on the unchanged conventional design, and in the second stage we used these findings to optimize the design and production process. The results of the first stage showed that the production of miniature, negative features was most problematic, regardless of materials or print settings. We determined that this is caused by an effect known as “false printing,” causing partial curing of surrounding layers. Therefore, in the second stage we presented a new design of the trocar that circumvents these effects by reducing the surface area per layer and creates more space surrounding the feature. With this new design, we succeeded in printing a miniature cannula with an open internal channel of 0.44 mm, and a functional, flexible valve. We evaluated the performance of this trocar on an eye phantom, which showed that it can be inserted and extracted from the eye without breakage. However, the measured insertion forces were 70% higher than from a commercial trocar, which can be attributed to the higher wall thickness and ribbed structure of the cannula.

Overall, in this work we have shown the potential of using SLA for the production of a miniature, functional trocar We have shown an approach in which we systemically test relevant 3D print settings, materials, and part dimensions in order to obtain the required functionality and accuracy for our device. Although vat photopolymerization techniques are reported to have one of the highest theoretical resolutions, there are still a number of practical limitations hindering the adaptation of these technologies for miniaturized, functional devices. Mapping these limitations can help manufacturers and engineers to improve these AM techniques and develop materials and workflows for improved accuracy for functional designs.

## Data Availability Statement

The raw data supporting the conclusions of this article will be made available by the authors, without undue reservation.

## Author Contributions

KL and MS contributed to the conception and execution of the initial design and experiments. KL designed, executed the optimized design, experiments, and wrote the first draft of the manuscript. KL, MS, and AS devised the structure of the manuscript. PB applied for funding. MS, AS, and PB revised the manuscript. All authors contributed to manuscript revision, read, and approved the submitted version.

## Funding

This project has received funding from the Interreg 2 Seas programme 2014–2020 co-funded by the European Regional Development Fund under subsidy contract No. 2S04-014.

## Conflict of Interest

The authors declare that the research was conducted in the absence of any commercial or financial relationships that could be construed as a potential conflict of interest.

## Publisher's Note

All claims expressed in this article are solely those of the authors and do not necessarily represent those of their affiliated organizations, or those of the publisher, the editors and the reviewers. Any product that may be evaluated in this article, or claim that may be made by its manufacturer, is not guaranteed or endorsed by the publisher.
