# Supplementary material for: Additive Manufacturing of a Miniature Functional Trocar for Eye Surgery
Source: Front Med Technol. 2022 Feb 17;4:842958. doi: 10.3389/fmedt.2022.842958 (PMC8891482; doi:10.3389/fmedt.2022.842958)
Supplement: Supplementary file 1 [file Data_Sheet_1.docx]

Supplementary Material

Figure S1

**
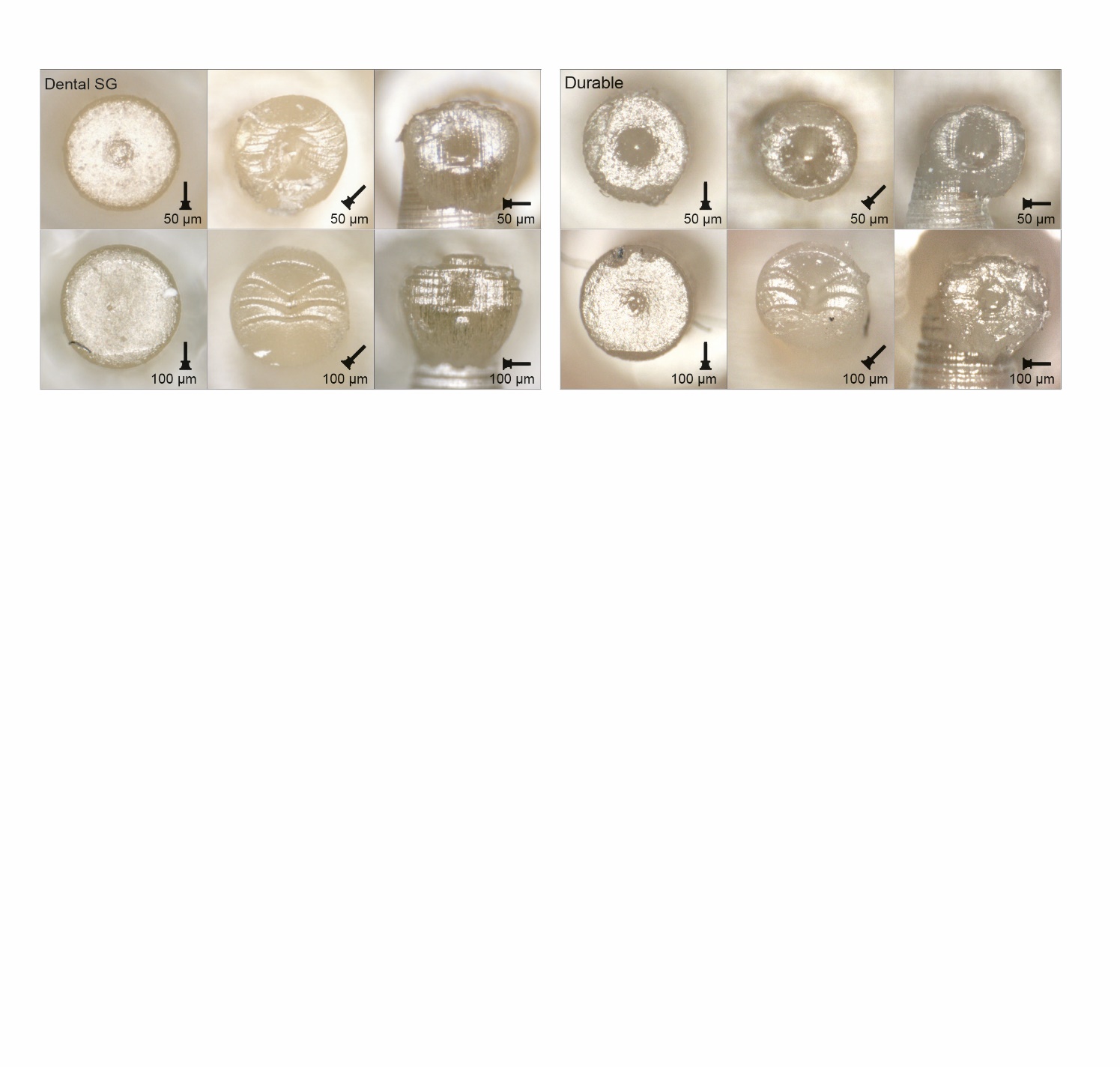
**

Figure S1. Example of the distal ends of the valves printed in Dental SG (left) and Durable (right), in different orientations and with different layer heights. For the cannulas in the 90º orientation, the support pillar is visible at the bottom.

Table S1

Table S1. Measurements of the outer channel of the 3D printed cannulas for the different build orientations and layer heights per material (n = 3). The lowest layer height was 50 µm for the materials Dental SG, Grey Pro and Durable, and 25 µm for Model. The highest layer height was 100 µm for each material.

|  | **Dental SG** | | | **Model** | | | **Grey Pro** | | | **Durable** | | |
| --- | --- | --- | --- | --- | --- | --- | --- | --- | --- | --- | --- | --- |
| Orientation | 0˚ | 45˚ | 90˚ | 0˚ | 45˚ | 90˚ | 0˚ | 45˚ | 90˚ | 0˚ | 45˚ | 90˚ |
| Lowest layer height (µm) | 997  ±14 | 965  ±11 | 1002  ±5 | 855  ±10 | 737  ±16 | 801  ±31 | 689  ±4 | 702  ±18 | 726  ±7 | 854  ±6 | 848  ±9 | 914  ±31 |
| Highest layer height (µm) | 1031  ±8 | 1025  ±2 | 1032  ±4 | 728  ±22 | 694  ±14 | 715  ±43 | 737  ±2 | 713  ±6 | 757  ±7 | 959  ±6 | 936  ±5 | 985  ±9 |

Figure S2

The negative surface area, or “openness” of the top surface of the valve was calculated as a percentage of the top surface. The images taken with the digital microscope were converted into vector drawings, and imported in the CAD software, which was then used to calculate the surface area of the vectors. If the printed thickness of the slits is larger than 10 µm, the valve will not be able to sufficiently prevent leakage. For the ideal slit size of 10 µm, the openness value corresponds to 0.84%. The calculated openness for all materials is given in Figure S4b. When the printed openness is close to the drawn openness, the results show a better accuracy. None of the printed valves had an openness close to the desired openness, which was to be expected based on the measurements of the slits. Durable (5.8%) and Elastic 50A (13.2%) show the best accuracy, since their printed openness is close to the as-drawn openness (5.9% and 13.9%, respectively). The valve printed in Flexible 80A showed the least openness, however this was because only the central opening of the cross slits had been printed open.


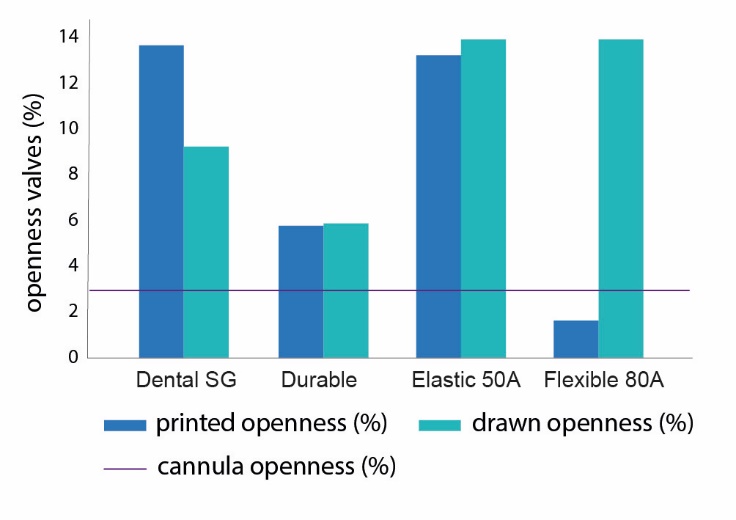


Figure S2. The percentage of openness of the valves as-printed compared to the openness of the valves as-drawn. As a reference, the openness of a 25G cannula is given as a percentage of the total valve surface (purple line).

Figure S3


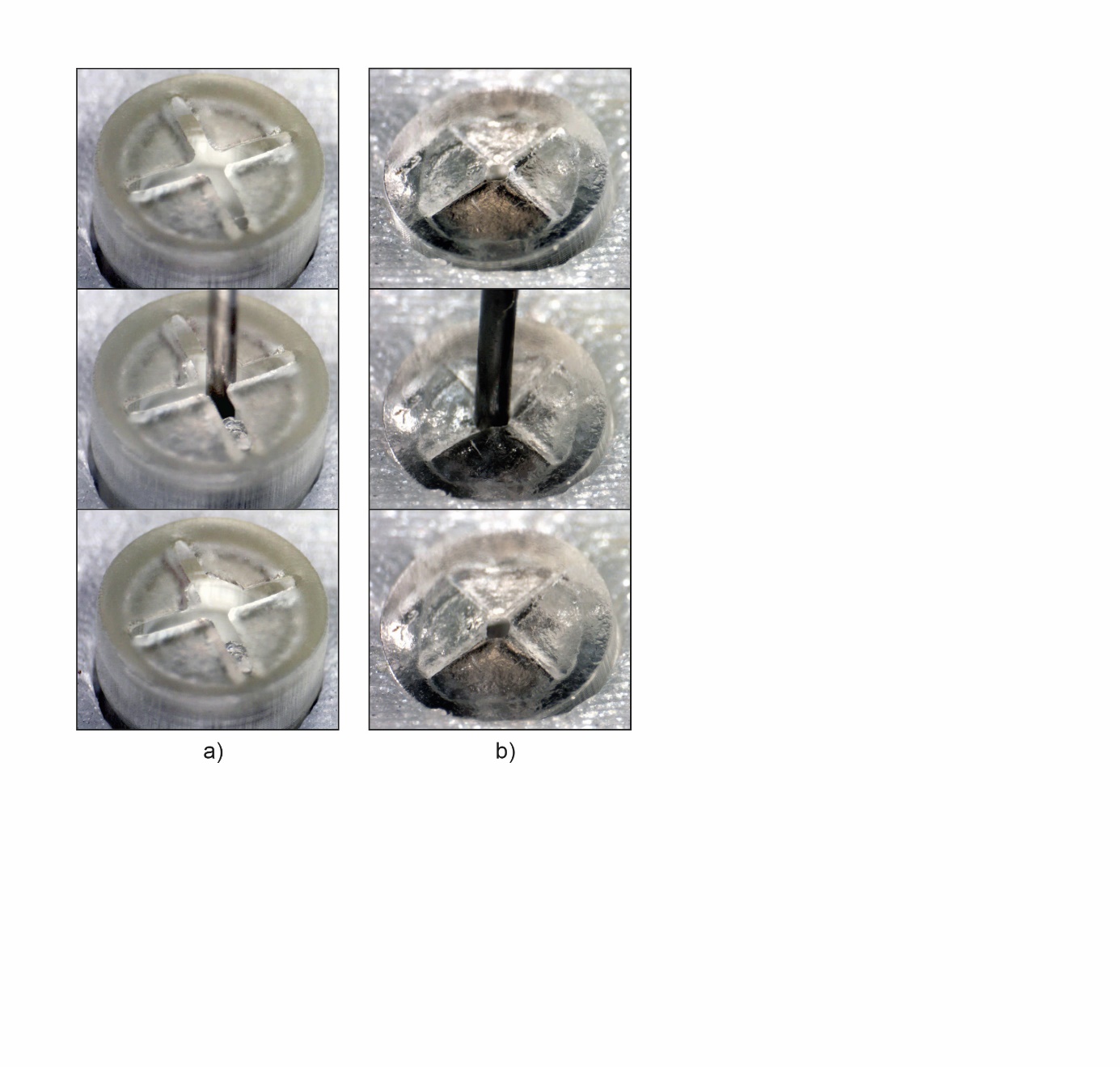


Figure S3. Results of the investigation into different materials and print settings for the valves. a) Example of a valve printed in Dental SG, showing the valve before, during, and after insertion of a needle. It can be seen that the valve flaps broke after insertion of the needle. b) Example of a valve printed in Flexible 80A, showing the valve before, during, and after insertion of a needle. It can be seen that the valve deforms, but does not break.
